# Hemicentral retinal vein occlusion in a patient with a history of coronavirus disease 2019 infection: a case report and review of the literature

**DOI:** 10.1186/s13256-023-04333-x

**Published:** 2024-02-11

**Authors:** Hamid Riazi-Esfahani, Reza Sadeghi, Mahdi Soleymanzadeh, Hossein Farrokhpour, Fatemeh Bazvand, Nazanin Ebrahimiadib, Elias Khalili Pour, Masoud Mirghorbani

**Affiliations:** Eye Research Center, Farabi Eye Hospital, Tehran University of Medical Sciences, Qazvin Square, Tehran, 1336616351 Iran

**Keywords:** COVID-19, Hemicentral retinal vein occlusion, Late manifestation, MTHFR, Mutation

## Abstract

**Background:**

Considering the various manifestations of coronavirus disease 2019 and its imperative importance in terms of the right clinical approach and early management, we sought to present a hemicentral retinal vein occlusion case, with a history of heterozygosity of *methylenetetrahydrofolate reductase* (*MTHFR*) genes and potential for clotting complications as a late manifestation of coronavirus disease 2019, and provide a brief review of reported retinal vein occlusion cases in patients with coronavirus disease 2019.

**Case presentation:**

A 35-year-old Iranian patient presented with a visual impairment in the left eye 4 months after recovering from coronavirus disease 2019. He reported a mild blurring of vision in the same eye a few days after admission due to coronavirus disease 2019. The ophthalmic evaluation was compatible with hemicentral retinal vein occlusion. Systemic and laboratory workups were negative except for borderline protein C activity, homocysteine levels, and heterozygosity of *MTHFR* genes. The patient was scheduled to receive three monthly intravitreal antivascular endothelial growth factor injections.

**Conclusion:**

We present a case of inferior hemicentral retinal vein occlusion case with an *MTHFR* mutation with sequential loss of vision 4 months after coronavirus disease 2019 to make clinicians aware of the possibility of late ocular coronavirus disease 2019 manifestations.

## Background

With the wide spectrum of organs impacted and the subsequent symptoms of coronavirus disease 2019 (COVID-19), devoting attention must be paid to the seemingly unimportant, yet potentially alarming, symptoms. Backed up by a robust body of evidence indicating the hypercoagulability state secondary to the cytokine storm, thromboembolic events associated with COVID-19 have been observed at several sites in the body [1]. The occurrence of central retinal vein occlusion (CRVO), considered an ocular emergency and potential threat to sight, is among these events that can lead to serious complications in the event of failure to diagnose.

Here, we present a unique case of a healthy 35-year-old Iranian patient with a history of mild blurring of vision in the left eye after admission due to COVID-19, which manifested later with more severe visual symptoms, and was diagnosed with hemicentral retinal vein occlusion (hemi-CRVO).

## Case presentation

**Fig. 1:**
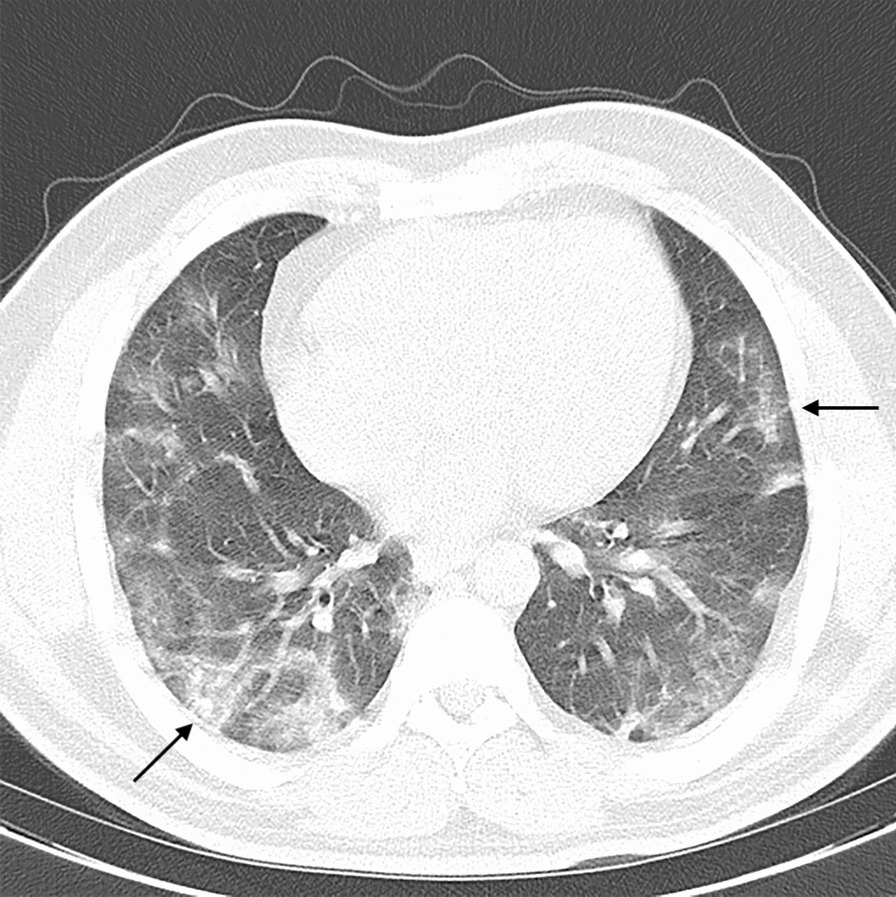
Chest CT scan shows bilateral multifocal subpleural and peribronchial ground-glass opacities (black arrows) compatible with COVID-19 disease

A 35-year-old Iranian male, with no previous history of hypertension, diabetes mellitus, immune-compromised diseases, or obesity, presented to the emergency department of a tertiary eye care center in Tehran, Iran with painless decreased vision in his left eye starting a week before the presentation. Familial and habitual history (for example, smoking) were unremarkable. The patient had been hospitalized with the diagnosis of COVID-19 4 months before the current ocular symptoms, confirmed by a positive SARS-Cov-2 polymerase chain reaction (PCR) test and a spiral chest computed tomography (CT) scan (Fig. 1). There was a history of mild blurry vision in his left eye that started acutely after COVID-19 infection. However, the vision was significantly lower in the current presentation. The patient did not seek an ophthalmic exam due to mild visual symptoms at the previous presentation. During hospitalization due to COVID-19, the patient received azithromycin, dexamethasone, ceftriaxone, and enoxaparin for 3 days.

**Fig. 2:**
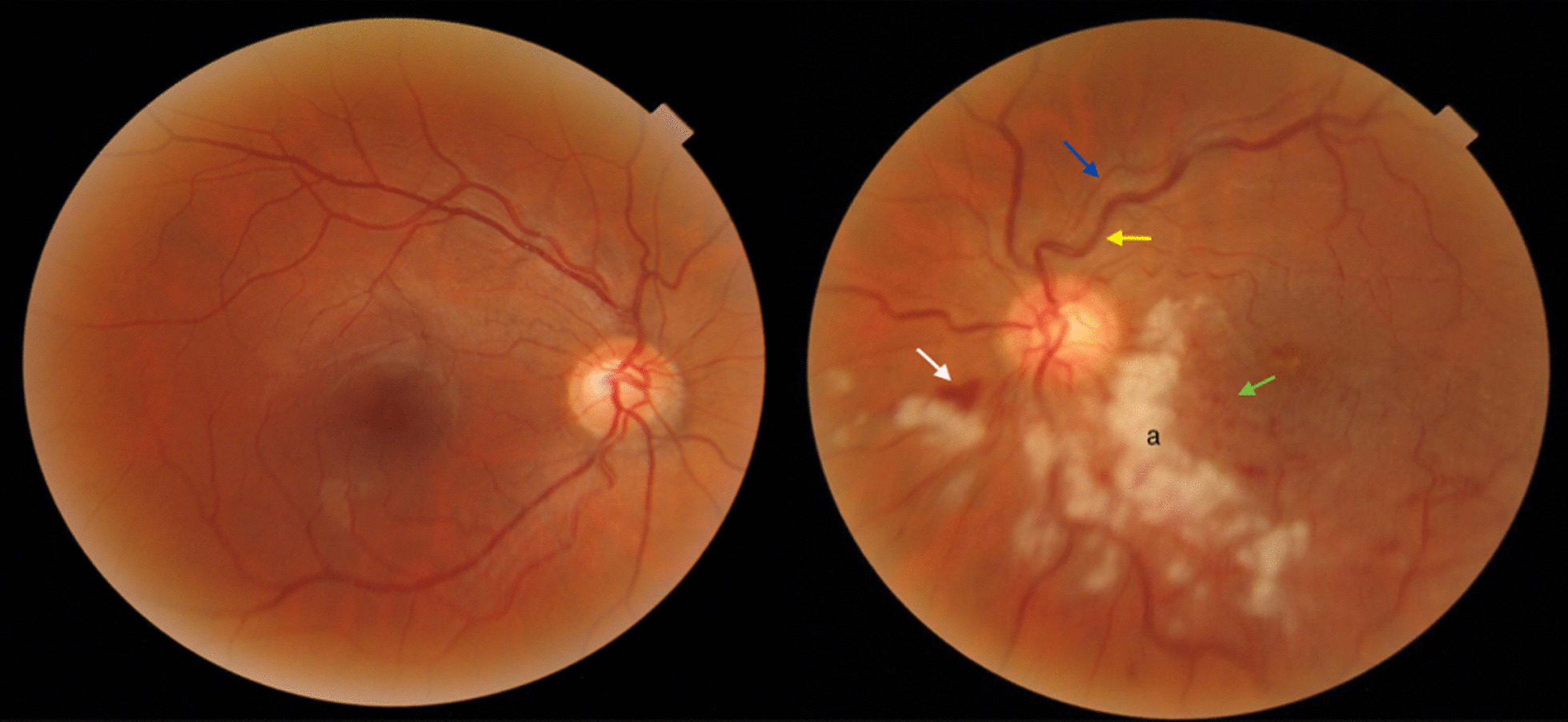
The color fundus photograph of the right eye is unremarkable. The left eye shows multiple cotton wool spots (**a**), flame-shaped hemorrhages (white arrow), and dot and blot hemorrhages (green arrow) in the inferior retina, which are compatible with inferior hemicentral RVO. Meanwhile, tortuosity and engorgement of veins (yellow arrow) and arteriolar narrowing (blue arrow) in supratemporal vascular branches are obvious

His best-corrected visual acuity (BCVA) in the right eye was 20/20, while it was reported as counting fingers (CF) at 2 m in the left eye. Ocular motility, pupillary reaction, intraocular pressure, and anterior segment examinations were normal. Fundus examination of the left eye showed multiple cotton wool spots, flame-shaped hemorrhages, and dot and blot hemorrhages along with the inferior retinal quadrants. Tortuosity and engorgement of veins and arteriolar narrowing in supratemporal vascular branches were also detected (Fig. 2).

**Fig. 3:**
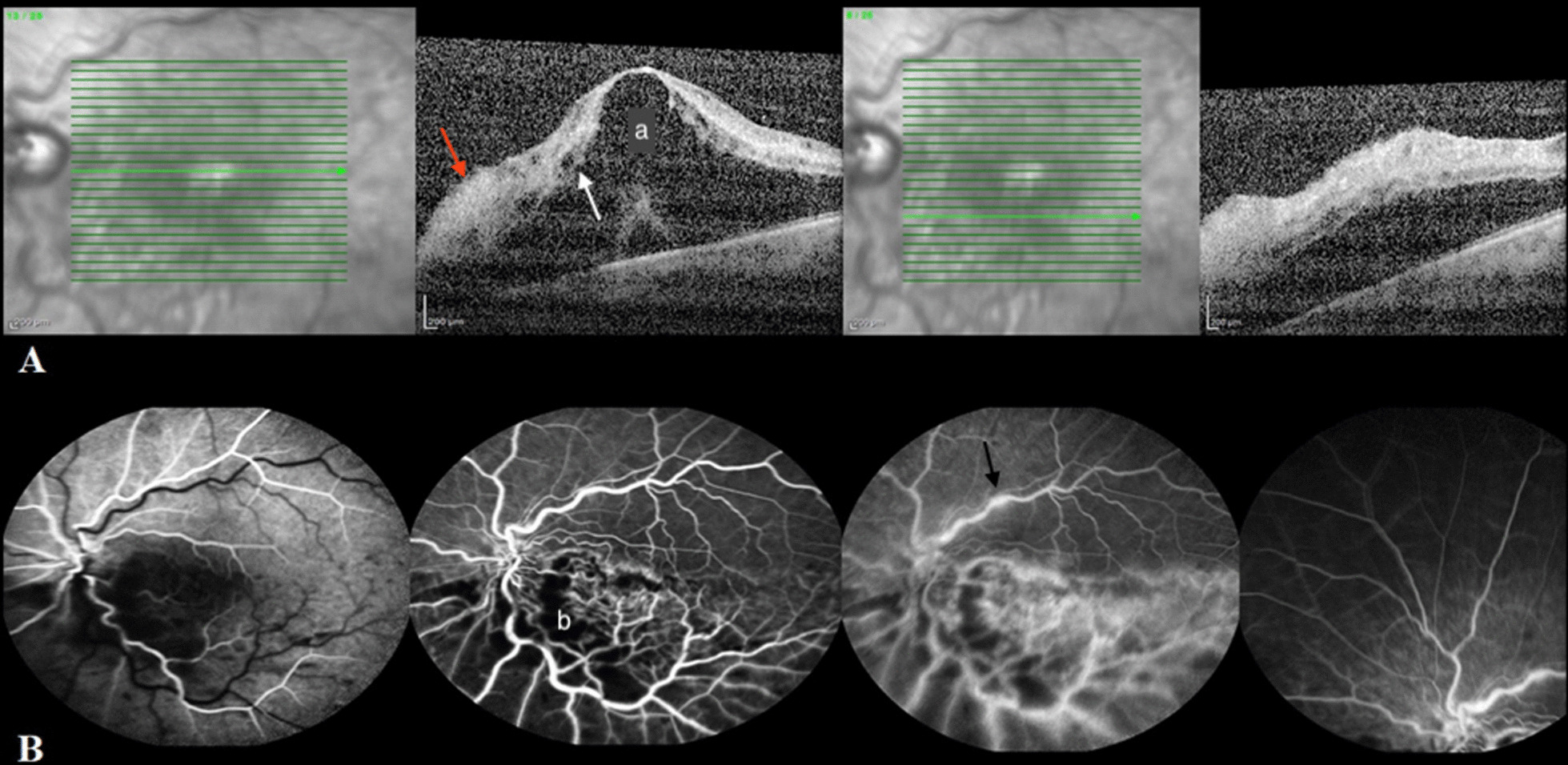
Spectral-domain optical coherence tomography (SD-OCT) and fluorescein angiogram (FA) of the left eye. **A** SD-OCT shows severe macular edema (**a**) and subretinal fluid, intraretinal cyst formation (white arrow), and hyperreflectivity of inner retinal layers (red arrow). **B** Nonperfusion areas (**b**) and vascular wall staining due to ischemia in the inferior retina and staining of a supratemporal branch of the retinal vein (black arrow) were detected in FA

Spectral-domain optical coherence tomography (SD-OCT) (Heidelberg Engineering, Heidelberg, Germany) of the left eye (Fig. 3A) demonstrated severe macular edema, subretinal fluid, intraretinal cyst formation, and hyper-reflectivity of inner retinal layers. Fundus fluorescein angiogram of the left eye (Fig. 3B) indicated nonperfusion areas and vascular wall staining due to ischemia in the inferior half of the retina, which was compatible with inferior hemi-CRVO with a probable ischemic nature. Additionally, tortuosity and staining of a few vessels superior to the fovea and staining of a supratemporal branch of the retinal vein without an adjacent nonperfusion area were detected.

Laboratory evaluations of the suspected hypercoagulability state were found normal, including activated partial thromboplastin time (aPTT), prothrombin time (PT), international normalized ratio (INR), lupus anticoagulant dilute Russell’s viper venom test (LAC dRVVT screen), LAC-sensitive PTT, antithrombin-III, lupus anticoagulant silica clotting time ratio (LAC SCT ratio), protein S antigen, anticardiolipin IgM and IgG, factor V Leiden PCR, and prothrombin (G20210A). Protein C activity and homocysteine level results were borderline. *Methylenetetrahydrofolate reductase* (*MTHFR*) gene assessment revealed heterozygosity for both *MTHFR* 677 and *MTHFR* 1298.

The patient was scheduled to receive three monthly intravitreal injections of antivascular endothelial growth factor (anti-VEGF) to treat RVO-related macular edema.

## Discussion and conclusion

Ocular manifestations of COVID-19 have been mainly related to the ocular surface, including chemosis, epiphora, conjunctival hyperemia, keratoconjunctivitis, pseudomembranous, and hemorrhagic conjunctivitis. [2] However, there have been reports of retinal involvement due to COVID-19 comprising uveitis, optic neuritis, neuroretinitis, paracentral acute middle maculopathy (PAMM), acute macular neuroretinopathy (AMN), papillophlebitis, cotton-wool spots, microhemorrhages along the retinal arcade on fundus photography, and hyperreflective lesions at the level of the ganglion cell and inner plexiform layers on optical coherence tomography (OCT) [3–5].

Herein, we report a healthy patient with a sequential history of blurred vision in his left eye following COVID-19. While the mild visual symptoms that were caused immediately after COVID-19 were resolved, inferior hemi-CRVO was developed 4 months later.

The rapid onset of painless visual loss during a short period entails several diagnoses, including, but not limited to, optic neuritis, retinal detachment or tear, diabetic retinopathy, retinal vasculitis disorders, ocular ischemic syndromes (OIS), and retinal vascular occlusions. Although the patient’s low age and unremarkable medical history favored optic neuritis or retinal detachment/tear as the more probable diagnosis, the history of COVID-19 favored inflammatory or hypercoagulable mechanisms, including vasculitis and RVO. Furthermore, the lack of precipitating symptoms, such as floaters, and risk factors made retinal detachments as a potential candidate less likely. The fundus photograph evaluation of the patient nominated diabetic retinopathy, hypertensive retinopathy, radiation retinopathy, and cytomegalovirus retinopathy as candidates next to the CRVO with the potential to cause the clinical image. However, the negative medical history of the patient and normal blood workup made them less likely to be the cause. Indeed, the features depicted in the SD-OCT, particularly the severe macular edema, subretinal fluid, intraretinal cyst formation, and hyperreflectivity of inner retinal layers, were highly suggestive of RVO as the primary diagnostic candidate. It is worth noting that some individual findings in the OCT could be found in diabetic retinopathy or OIS. However, the combination of SD-OCT and fundus characteristics strongly pointed toward CRVO. Subsequently, the nonperfusion area detected in the inferior fluorescein angiography confirmed hemi-CRVO as the final diagnosis.

The prognosis of CRVO is significantly associated with the disorder being ischemic or nonischemic, the presenting VA, and the occurrence of later complications. The main factors contributing to the poor prognosis are macular ischemia and the future chance of anterior segment neovascularization or neovascular glaucoma [6]. Most of the patients with CRVO with visual acuity (VA) > 20/200 will return to normal VA. On the other hand, patients with ischemic CRVO present mostly with a VA < 20/200 and have a very poor prognosis. In a study of patients with a presenting visual VA < 20/200, improvement occurred in only 20% [7]. Despite this low rate, it is of vital importance to diagnose macular edema in these patients and target it with anti-VEGF therapies to lower the residual effects as much as possible. Since the diagnosis of the presented patient in the current study was more likely to be the ischemic type hemi-CRVO due to the low presenting visual acuity (VA) and the evidence observed in the fluorescein angiography, the prognosis is not expected to be high, and there is a chance that the visual impairment may be permanent.

Recently, it has been shown that COVID-19 can lead to systemic vasculitis. Type III hypersensitivity, caused by the deposition of the immune complexes, can mediate an inflammatory response through cytokine cascades. Moreover, the out-of-control release of a large variety of proinflammatory cytokines, a cytokine storm, results in endothelial damage. The procoagulant condition caused by the systemic vasculitides and cytokine cascades can cause venous and arterial thrombosis [8].

This is the second case of an *MTHFR* mutation in a patient with COVID-19 and CRVO. Staropoli *et al*. described a case of a 15-year-old boy with an intriguing initial presentation of CRVO and concurrent asymptomatic COVID-19 [9]. The homocysteine levels were not increased in the study by Starpoli *et al*., in contrast to the moderately increased levels in the present report. Although the normal homocysteine levels make establishing a hypercoagulability state due to *MTHFR* mutation questionable to some extent, it does not entirely disregard it. Interestingly, Gao *et al*. investigated the *MTHFR* C677T (presented in this report) mutation in patients with CRVO and concluded this mutation is a potential susceptible factor for CRVO [10]. The association between the *MTHFR* mutation and elevated homocysteine levels with CRVO has been shown by a high volume of evidence [11]. However, the direct role of *MTHFR* mutation in the occurrence of CRVO remains inconsistent, as other studies have revealed that this mutation is not an independent risk factor for CRVO [12, 13]. All in all, considering the emerging body of evidence, there might be at least a partial, highlighting the need for future studies to address this gap in future studies.

A systemic evaluation for RVO in younger patients (less than 55 years) without any related risk factors may be necessary to detect inflammatory, infectious, or hypercoagulability etiologies [14]. Although our patient was heterozygote for both *MTHFR* 677 and 1298, the role of the *MTHFR* gene remains controversial as a risk factor for RVO [13]. However, the presence of heterozygosity for both types of *MTHFR* genes, borderline protein C activity, and homocysteine levels may have facilitated the prothrombotic events in this patient with COVID-19.

Reported RVO cases in patients with COVID-19 are presented in Table 1 in chronological order. In contrast to the present case, the previously reported patients with CRVO with a history of COVID-19 were mainly older than 40 years and had underlying diseases.

**Table 1:** Reported retinal vein occlusion (RVO) cases in patients diagnosed with COVID-19 disease

Authors (time of publication)	Demographics	Comorbidities	Time interval with COVID-19 diagnosis	Method of COVID-19 diagnosis	Severity of COVID-19*	Ocular manifestation	Positive coagulopathy studies **
Insausti *et al*. [17] (July 2020)	40 y/o male	Negative	6 weeks before	ELISA	Mild	CRVO in left eye	d-Dimer (672 μg/L), Fibrinogen (451 mg/dL)
Sheth *et al*. [18] (October 2020)	52 y/o male	Negative	10 days before	PCR	Moderate	Inferior HCRVO with superonasal BRVO in left eye	Unremarkable
Gaba *et al*. [19] (October 2020)	40 y/o male	Hypertension, morbid obesity, deep vein thrombosis	3 days before	PCR, HRCT	Severe	Bilateral CRVO	d-Dimer (> 20000 µg/L)
Invernizzi *et al*. [20] (November 2020)	54 y/o female	Negative	5 days before	Not mentioned	Mild	CRVO in right eye	PT (13.8 s) INR (1.27) Fibrinogen (682 mg/dL)
Walinjkar *et al*. [21] (November 2020)	17 y/o female	Polycystic ovaries	23 days before	HRCT, serology (IgG)	Mild	CRVO in right eye	Unremarkable
Yahalomi *et al*. [22] (December 2020)	33 y/o male	Negative	5 to 2 weeks before	Serology (IgG)	Mild	CRVO in left eye	Slightly abnormal fibrinogen and d-Dimer
Monferrer *et al*. [23] (December 2020)	59 y/o female	DM	18 days after ocular manifestations	PCR	Mild	Bilateral CRVO	Thrombocytopenia (126 × 109/L)
Lorca *et al*. [24] (December 2020	30 y/o female	DM (maturity onset)	1 month after discharge	Not mentioned	Moderate	Bilateral CRVO	Substantial increases value of d-dimer, ferritin, fibrinogen, and platelets
Duff *et al*. [16] (February 2021)	74 y/o female	Hyperlipidemia	Concurrent	Not mentioned	Mild	Infratemporal BRVO in left eye; CME occurred 3 months later	Unremarkable
Finn *et al*. [15] (March 2021)	32 y/o male	Negative	2 months before	PCR and ELISA	Mild	inferior HCRVO in right eye	Unremarkable
Raval *et al*. [25] (April 2021)	39 y/o male	Negative	7 days before	PCR	Mild	CRVO in right eye	Unremarkable
Venkatesh *et al*. [26] (May 2021)	56 y/o female	DM	Asymptomatic	Serology (IgG)	Asymptomatic	CRVO in left eye	d-Dimer (707 μg/L), PT (14.8 s) INR (1.18)
Al-Abri *et al*. [27] (July 2021)	33 y/o male	Negative	6 month prior to the presentation ***	Not mentioned	Mild	CRVO in left eye	Unremarkable
Staropoli *et al*. [9] (April 2022)	15 y/o male	Homozygous for MTHFR	Concurrent	PCR	Asymptomatic	CRVO in left eye	Homozygous MTHFR Normal homocysteine levels
Modjtahedi *et al*. [28] (April 2022)	A cohort of 16 patients (among 432,515 patients with COVID-19)	Not determined	From 2 weeks before to 26 weeks after diagnosis	PCR	N/A	Not determined on the basis of every patients	N/A

*BRVO* branch retinal vein occlusion, *CME* cystoid macular edema, *CRVO* central retinal vein occlusion, *DM* diabetes mellitus, *ELISA* enzyme-linked immunosorbent assay, *HCRVO* hemi – central retinal vein occlusion, *HRCT* high resolution computed tomography, *INR* international normalized ration, *PCR* polymerase chain reaction, *PT* prothrombin time

^*^Mild = treated as outpatient; moderate = hospitalized; severe = ICU admit

^**^abnormal ranges: d-Dimer > 500 μg/L; fibrinogen > 400–500 mg/dL (depending on laboratory references); PT > 13.5 s; INR > 1.11; platelet count < 150 × 10^9^/L

^***^Patients symptoms started after administration of COVID-19 (Pfizer-BioNTech) vaccine

The marked delay in the presentation was another difference between our case and the existing literature. However, there have been patients experiencing CRVO after a certain time from COVID-19. Finn *et al*. reported a 32-year-old previously healthy man diagnosed with COVID-19 who was referred for ophthalmic care due to blurring of vision in the superior visual field. One month later 2 months after the onset of COVID-19), there were worsening visual symptoms, and he was diagnosed with hemi-CRVO. All laboratory workups were unremarkable, including PT, PTT, CBC, anticardiolipin antibodies, lupus anticoagulant, factor V Leiden, and protein C [15]. Similarly, Duff *et al*. reported a healthy 74-year-old female patient who originally appeared with blurred vision in her left eye with symptomatic COVID-19. The patient was diagnosed with branch RVO, which did not immediately require treatment. She came back with deteriorating vision 3 months later and was diagnosed with cystoid macular edema (CME) due to the vein occlusion, which was treated with an intravitreal dexamethasone implant [16]. Similar to these two cases, our patient presented with a sequential visual symptom that progressed. We hypothesize that an ocular vascular accident in our patient might have occurred early after COVID-19 due to cytokine storm and consecutive vasculitis and/or thromboembolic events that progressed toward hemi-CRVO. Progression of ischemia or late-onset CME formation may cause later visual loss.

Here, we report the case of a relatively young patient with sequential visual loss 4 months after COVID-19 who was diagnosed with inferior hemi-CRVO. The probable contributing factor was the high risk of clotting complications in patients with COVID-19. This study underscores the possibility of late ocular COVID-19 complications despite the mild and seemingly unimportant nature of the primary symptoms. Further investigations are needed to establish the causal association and address the underlying mechanisms.

## Data Availability

The datasets used in the current study are available upon reasonable request.

## Abbreviations

AMN: Acute macular neuroretinopathy
Anti-VEGF: Anti-vascular endothelial growth factor
aPTT: Activated partial thromboplastin time
CF: Counting fingers
CME: Cystoid macular edema
COVID19: Corona virus 2019
CRVO: Central retinal vein occlusion
INR: International normalized ratio
LAC SCT ratio: Lupus anticoagulant Silica Clotting Time ratio
MTHFR: Methylenetetrahydrofolate reductase
OIS: Ocular ischemic syndrome
PAMM: Paracentral acute middle maculopathy
PCR: Polymerase chain reaction
PT: Prothrombin time
RVO: Retinal vein occlusion
SD-OCT: Spectral-domain optical coherence tomography
